# Factors predicting outcome in cervical lymph node tuberculosis: insights from a Tunisian case series

**DOI:** 10.12688/f1000research.164097.1

**Published:** 2025-05-21

**Authors:** Maissa Lajhouri, Selima Jouini, Yosra Ammar Mnejja, Azza Mediouni, Rihab Lahmar, Houda Chahed

**Affiliations:** 1 Department of otorhinolaryngology- Head and Neck Surgery, La Rabta University Hospital Center, Tunis, 1007, Tunisia

**Keywords:** tuberculosis, lymphadenopathy, tuberculous lymphadenitis, Mycobacterium Bovis, outcome.

## Abstract

**Background:**

Tuberculosis remains a significant public health issue in Tunisia. This study aimed to describe the epidemiological, clinical, and therapeutic characteristics of cervical lymph node tuberculosis and identify factors influencing outcomes.

**Methods:**

A retrospective study was conducted over a 3-year period in the ENT department at La Rabta Hospital, Tunis. Diagnosis was based on histopathological evidence, and disease progression was categorized as favorable (treatment <9 months, no additional surgery) or unfavorable (treatment >9 months and/or supplementary surgery).

The study population was divided into two groups based on the outcome nature, and analytical analysis was performed to assess factors influencing outcomes

**Results:**

The study included 102 patients (32 men and 70 women), with a median age of 34.5 years (range: 8-83 years). Most patients (78.4%) had no significant medical history or known HIV infection. Thirty-nine patients (38.2%) had a history of consuming raw milk. In 65 cases (63.7%), lymph node size exceeded 3 cm. Hypoechogenicity (53.9%) and necrosis (40.1%) were the most common findings on ultrasound and CT scan, respectively. The initial diagnostic approach included adenectomy (56.8%), lymph node dissection (8.9%), and drainage of cold abscesses (34.3%). All patients received an initial four-drug antituberculosis regimen. Ethambutol treatment was extended beyond 2 months in 65 cases (63.7%). Fifty-six patients (54.9%) had a favorable outcome.

Factors associated with a favorable outcome included intact skin, complete initial lymph node dissection, favorable progress at 2 months, and prolonged ethambutol therapy.

**Conclusions:**

The management of lymph node tuberculosis remains challenging, especially with insufficient bacteriological confirmation. Regional epidemiological factors should be considered. The role of surgery is crucial; however, further standardization is needed to optimize patient outcomes.

## Introduction

Tuberculosis continues to be a major health problem, with an incidence rate of over 10 million cases per year worldwide. ^1^ This disease causes significant morbidity and has a considerable mortality rate, estimated at around 1.3 million deaths, making tuberculosis the second most lethal infectious disease after COVID-19 in 2022. ^1^

Lymph node tuberculosis is the most common manifestation of extrapulmonary tuberculosis, with the cervical area being the most frequently involved. ^2^ Cervical lymph node tuberculosis can present real diagnostic and therapeutic challenges due to its paucibacillary nature and the increasing rate of resistance to antituberculosis drugs.

The aim of this study was to describe the epidemiological, clinical, and therapeutic features of cervical tuberculous lymphadenitis and to evaluate the potential factors influencing the course of the disease.

## Methods

### Study design and participants

This is a retrospective study conducted over a 3-year period, from 2019 to 2021. We reviewed clinical data from patients with cervical tuberculous lymphadenitis treated in the Head and Neck Surgery Department at La Rabta Hospital in Tunis. The diagnosis of tuberculous lymphadenitis was based on histopathological findings in all patients.

Initially, all patient records diagnosed with cervical tuberculous lymphadenitis were extracted. Patients who met the exclusion criteria were subsequently removed from the study. The exclusion criteria were as follows:

• Patients with poor adherence to treatment
• Patients who were not fully followed up in our department

The minimum follow-up period was 12 months after the completion of treatment.

### Evolution assessment

The progression was assessed based on two criteria: the duration of treatment and the need for a second surgical procedure to complete the treatment. These two criteria were used to divide the population into two groups: those with a favorable outcome (G1), defined as a treatment duration of less than 9 months without the need for surgical treatment and a lymph node size of less than 1 cm at the end of treatment, and those with an unfavorable outcome (G2), if the treatment lasted more than 9 months and/or if a second surgery was required.

The limit of 9 months was chosen to assess prognosis because, in some cases, the treatment was maintained for more than 6 months as imaging was not immediately available to control the lymph node size, or in the presence of residual adenopathy.

Recurrence was defined as the reappearance of tuberculous lymphadenitis or an increase in the size of lymphadenopathy after the completion of treatment, confirmed by bacteriological, molecular, cytological, or histopathological evidence. The minimum period after treatment completion required to define recurrence was 6 months of remission.

### Statistical methods

*Data analysis*

The clinical profiles of the enrolled patients were described using descriptive statistics, including mean values, standard deviations, medians, and interquartile ranges for continuous variables, and proportions for categorical variables.

An analytical study was conducted to compare two groups of patients to identify factors potentially associated with favorable or unfavorable disease progression. Epidemiological, clinical, biological, radiological, and therapeutic factors were evaluated.

*Univariate analysis*

- Comparisons between two qualitative variables were performed using Pearson’s chi-squared test, or Fisher’s exact test when assumptions for the chi-squared test were not met.
- Comparisons between one qualitative variable and one quantitative variable were conducted using:
  ○ Student’s t-test for variables following a Gaussian distribution (normality tested using the Shapiro-Wilk test).
  ○ Non-parametric tests (e.g., Mann-Whitney U test) for variables not following a Gaussian distribution.
- Odds Ratios (OR) were calculated for qualitative variables to estimate risk factors.

*Multivariate analysis*

A multivariate analysis was conducted using a logistic regression model (or Cox regression for survival data). Variables with p < 0.2 in univariate analysis were included. The results were expressed as Odds Ratios (OR) with 95% Confidence Intervals (CI).

Statistical analysis was performed using SPSS version 22. A p-value < 0.05 was considered statistically significant.

## Results

### Descriptive analysis

A total of 161 cases of cervical lymph node tuberculosis were initially diagnosed, but only 102 patients were ultimately included.

The group included 32 men and 70 women, with a sex ratio of 0.46. The median age was 34.5 years (range: 8-83). Sixty-one patients (60%) were under the age of forty. Only 22 patients (21.5%) had a special medical history (hypertension, diabetes, pulmonary tuberculosis, autoimmune diseases: Systemic lupus erythematosus, Behçet’s disease, Crohn’s disease, thyroiditis). No cases of known human immunodeficiency virus (HIV) infection were reported. Thirty-nine patients (38.2%) had a history of consuming raw milk.

Tuberculosis was discovered during the exploration of systemic symptoms in 59 patients (57.8%), while 40 patients (39.2%) presented with neck swelling as the first complaint. Two patients were already under treatment for pulmonary and one patient for osteoarticular tuberculosis, and cervical lymphadenitis was detected on radiological examination.

**Figure 1.**
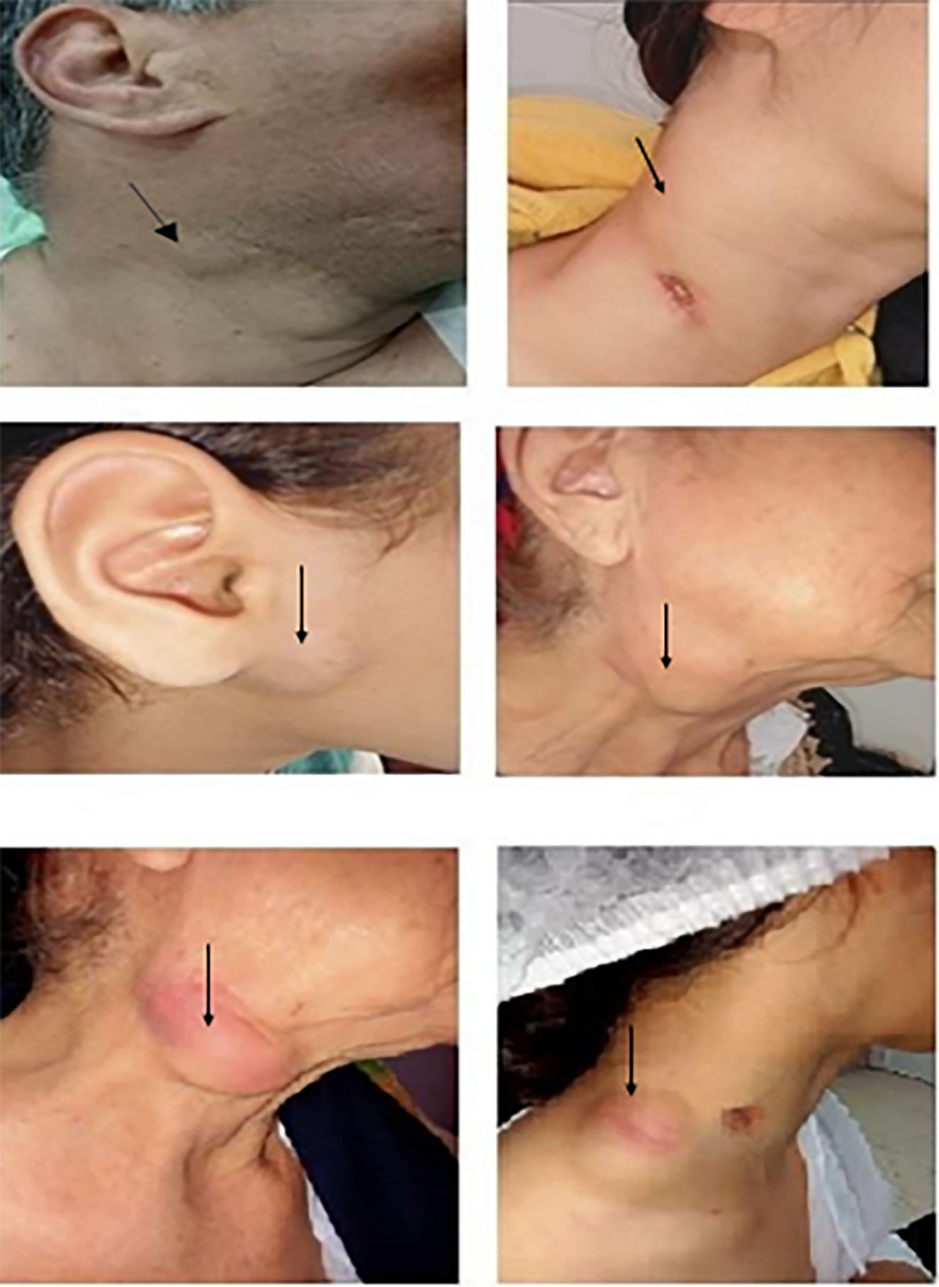
Clinical Features of Tuberculous Lymphadenopathy.

On physical examination, 86 patients (84.3%) had multiple palpable nodes, mostly unilateral (76 patients: 74.5%). The most affected lymph node levels were II and III (94 patients: 92.1%). The median size of the lymph nodes was 3 cm (range: 1.5 cm to 7.5 cm). In 65 cases (63,7%), lymph node size exceeded 3 cm. The skin was inflammatory in 28 cases (27.4%) and a cutaneous sinus tract was present in 9 cases (8.8%) ( Figure 1).

Biological tests revealed anemia in 25 patients (24.5%) and lymphopenia in 22 patients (21.5%). HIV testing was performed in only three patients, and all tests were negative.

The tuberculin skin test was performed in 88 patients (86.2%). An induration or erythema diameter greater than 10 mm was observed in 75 patients.

**Figure 2.**
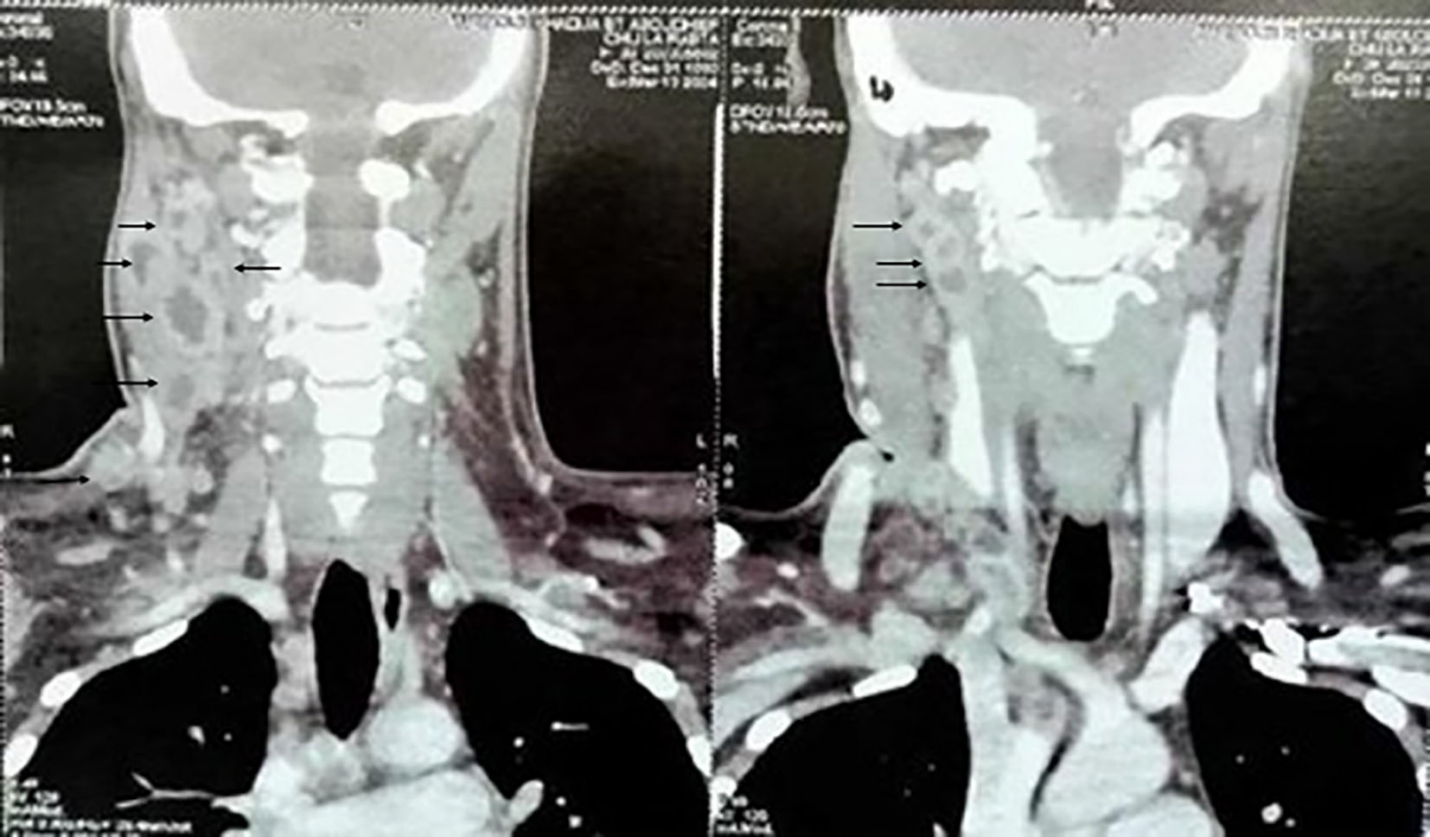
Multiple Necrotic Cervical Lymphadenopathies on CT Scan.

All patients had cervical ultrasound (US) and/or computed tomography (CT) of the neck and thorax. The most prevalent radiological features were hypo echogenicity on US (53.9%) and necrosis on CT (40.1%) ( Figure 2).

Imaging revealed the involvement of extra cervical lymph nodes in 22 patients (21.5%), particularly in the mediastinum (15 patients), and associated pulmonary tuberculosis in three patients.

Fine needle aspiration (FNA) was performed in 85 patients (83.3%). Epithelioid granulomas with necrosis were observed in 33 patients.

Microbiological testing was performed in 21 patients (20.6%). It revealed the presence of Acid-fast Bacilli on direct microscopic examination in 6 patients, but mycobacterium tuberculosis cultures were negative in all cases.

Molecular tests using the Xpert/MT PCR technique were carried out in 21 patients and were positive for *Mycobacterium tuberculosis* in 16 patients. Intermediate resistance to rifampicin was found in 7 patients.

**Figure 3.**
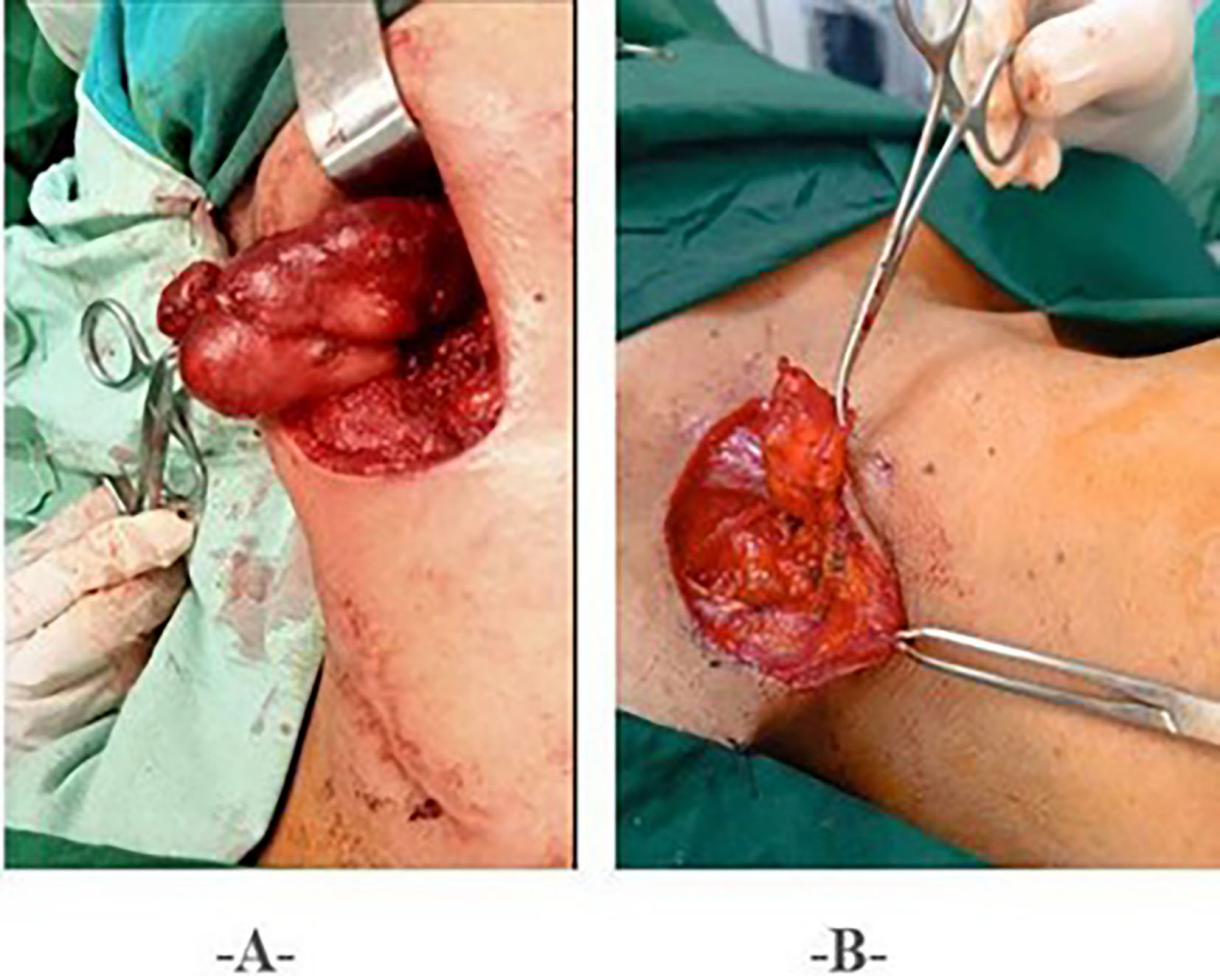
Surgical Procedures. • 3A: Lymph Node Dissection. • 3B: Lymphadenectomy.

Surgery associated with histopathological study was performed in all patients. The surgical procedures consisted of lymphadenectomy in 58 patients (56.8%), lymph node dissection in 9 patients (8.9%), and drainage of abscess with curettage/or fistula excision in 35 patients (34.3%) ( Figure 3).

A four-drug regimen (isoniazid, rifampicin, pyrazinamide, ethambutol) was systematically administered for two months. The following two-drug regimen (mainly Isoniazid + Rifampicin) was prescribed.

Ethambutol was maintained for more than two months, with an average duration of 4 months (range: 2–12 months) in 65 patients (63.7%).

Eleven patients received second-line antituberculosis drugs, consisting of fluoroquinolones.

By the second month of treatment, compared to the initial size of the lymphadenopathy, clinical improvement was observed in 51 patients (50%), stable lymph node size in 32 patients (31.3%), and a paradoxical reaction in 15 patients (14.7%).

The outcome was considered favorable in 56 patients (G1) and unfavorable in 46 patients (G2).

In G2, two patients required surgery with a total treatment duration of less than 9 months, 22 patients received anti-tuberculous treatment for more than 9 months, and the remaining 22 patients underwent surgery along with prolonged treatment.

Tuberculosis recurrence was observed in 12 patients (11.7%), with a median delay of 12 months (range: 7–63 months). These patients were predominantly G2 (11 patients: 91.6%).

### Statistical analysis

Univariate analysis (Table 1 Underlying data ^3^) revealed that the epidemiological characteristics and medical history were not significantly associated with any specific disease progression.

Analysis of physical examination data showed that pathological skin conditions (inflammatory skin/fistula) were significantly associated with unfavorable disease progression. No biological criteria were found to influence the outcome. Regarding imaging data, significant association was found between lymph node necrosis on CT and unfavorable disease progression. When the initial procedure involved abscess drainage, patients were more likely to experience an unfavorable outcome. However, if the initial procedure was lymph node dissection, patients were twice as likely to have a favorable outcome. The presence of intermediate resistance to rifampicin did not influence disease progression. Prolonged ethambutol prescription beyond the first two months of treatment was significantly associated with a favorable disease outcome.

Patients showing improvement by the second month were more likely to achieve a favorable outcome. Multivariate analysis ( Tables 2 and 3) revealed that pathological skin conditions, necrosis on CT, abscess drainage, occurrence of a paradoxical reaction, and lack of improvement by the end of the second month of treatment were independently associated with an unfavorable outcome. On the other hand, healthy skin adjacent to the lymph nodes, initial lymph node dissection, favorable evolution at the end of the second month of treatment, and prolonged ethambutol prescription beyond two months were factors independently associated with a favorable disease outcome.

**Table 2.** Multivariate Analysis of Factors Associated with Unfavorable Outcomes.

Factors associated with unfavorable evolution	P	Odds Ratio	95% Confidence Interval	
			Lower	Higher
Pathological adjacent skin	**0.024**	3.496	1.177	10.380
Necrosis on cervical CT scan	**0.044**	2.406	1.024	5.651
Drainage of a cold abscess	**0.029**	3.306	1.132	9.653
Paradoxical reaction (end of the second month)	**0.002**	9.633	2.285	40.613
Stagnation (end of the second month)	**0.000**	11.776	3.591	38.617

**Table 3.** Multivariate Analysis of Factors Associated with Favorable Outcomes.

Factors associated with favorable evolution	P	Odds Ratio	95% Confidence Interval	
			Lower	Higher
Healthy adjacent skin	**0.006**	3.637	1.452	9.113
Lymph node dissection	**0.030**	4.069	1.146	14.442
Improvement (end of the second month)	**0.003**	19.482	2.735	85.434
Prolonged ethambutol treatment	**0.002**	4.339	1.697	11.093

All data supporting these findings are publicly available through Zenodo (DOI: 10.5281/zenodo.15351592) ^3^

## Discussion

Tunisia is classified as a country with intermediate tuberculosis endemicity, with an estimated annual incidence between 100 and 299 cases per 100,000 inhabitants. ^1^ This relatively high incidence, coupled with increasing challenges in disease management, particularly in cases of cervical lymph node tuberculosis, highlights the importance of addressing this pathology.

### Descriptive analysis

Among the 102 study participants, most of the patients (61, or 60%) were under the age of 40 years. This finding is consistent with several published reports. ^2, 4, 5^ Furthermore, like other studies, a female predominance was observed, in contrast to the clear male predominance in pulmonary tuberculosis. ^2, 4^ The reasons for this female prevalence remain unexplained.

Some patients had autoimmune diseases, including Crohn’s disease. Certain treatments, such as tumor necrosis factor (TNF) antagonists, are now known to affect the antituberculosis immune response. ^6, 7^

The HIV-positive population was not represented in our study, as individuals with known HIV infection and tuberculous lymphadenitis are followed up in the infectious disease department at our institution. Moreover, only a few patients were tested for HIV. No conclusions can be drawn regarding HIV infection from our series, although many studies have reported a low HIV positivity rate in patients with tuberculous lymphadenopathy. ^2, 5, 8– 10^

In our study, a significant percentage of participants (38.2%) reported raw milk consumption, similar to other studies, suggesting that *Mycobacterium Bovis* strains may contribute to tuberculous lymphadenitis in certain regions. ^4, 11, 12^

Most patients had unilateral and multiple lymph node involvement. In their studies, Mathiasen et al. and Qian et al. reported unilateral involvement in most cases. ^10, 13^ However, cervical tuberculous lymphadenitis can be unilateral or bilateral, and all lymphatic levels of the neck can be affected.

Although in 32.3% of cases, FNA revealed epithelioid granulomas with necrosis, features highly suggestive of tuberculosis, all patients were treated based on histopathological findings indicative of tuberculosis. This approach could be explained by the low positivity rate of bacteriological tests in our study, the concern of misdiagnosing a malignancy, and, in some cases, the need for surgery in patients with abscesses or sinus tract formation.

Despite their high sensitivity, cytology and histopathology lack specificity for extrapulmonary tuberculosis. These methods detect granulomas that can also be present in other diseases, such as nontuberculous mycobacterial infections. ^14^ FNA is preferred as a cost-effective, safe and rapid method, particularly in endemic countries with limited resources. ^14, 15^ However, diagnosing tuberculosis solely based on cytological or histopathological criteria should be approached with caution and supported by other clinical evidence, such as positive results from the tuberculin skin test.

The isolation of *Mycobacterium tuberculosis* remains the gold standard for a confirmatory diagnosis of tuberculous lymphadenitis, either through culture or by PCR testing of a sample from an affected lymph node. ^16^

The culture positivity rate was low in our study. In fact, the rate of culture positivity in lymph node tuberculosis ranges from 18% to 62%. ^16^ The Xpert® MTB/RIF assay was the only molecular test performed, conducted in 20.6% of cases, likely due to availability issues. This targeted PCR technique, by detecting the Mycobacterium tuberculosis complex, provides a rapid diagnosis with greater sensitivity than culture and high specificity. It also detects the rifampicin resistance gene, which was found in 7 of our patients (6.8%). Other multiplex PCRs, detecting more resistance mutations on target genes, exist but are unavailable in hospital laboratories in Tunisia. Next-generation sequencing, which enables the simultaneous sequencing of multiple genes, is becoming increasingly available in many countries. The widespread adoption of these techniques in resource-constrained countries could be crucial for implementing more effective anti-tuberculosis therapy. ^14, 17^

Treatment of active tuberculosis involves a well-established two-phase approach: an initial two-month, four-drug regimen (Isoniazid, Rifampicin, Pyrazinamide, Ethambutol) followed by a maintenance phase with a two-drug regimen (Isoniazid, Rifampicin). The World Health Organization (WHO) recommends a treatment duration of six months for lymph node tuberculosis caused by sensitive strains. However, the treatment duration may extend beyond this period, with many studies reporting durations exceeding six months. ^16, 18, 19^ There remains controversy regarding the treatment duration for tuberculous lymphadenitis, as the course of the disease is unpredictable and there are no clear criteria for assessing cure. ^19^

In Tunisia, the official national practice guidelines recommend a treatment duration of six months, consistent with WHO guidelines. A Maghrebian recommendation published in 2015, considering the high incidence of *Mycobacterium Bovis* among cattle and the high consumption of raw milk products, suggested that the treatment duration could be extended to 12 months. It also recommended a prolonged prescription of ethambutol from 1 to 4 months following the initial four-drug regimen for patients with persistent tuberculous lymphadenitis after initial surgery, as *Mycobacterium Bovis* is naturally resistant to pyrazinamide. ^20^

*Mycobacterium Bovis*, present in unpasteurized milk and dairy products, is thought to reach the superior and anterior cervical lymph nodes through micro-ulcerations in the buccal mucosa. ^11, 12^

In their Tunisian series, Ghariani et al. identified *Mycobacterium Bovis* as the strain responsible for tuberculous lymphadenopathy in 76% of bacteriologically diagnosed cases (79 cases). ^9^ However, while the prevalence of *Mycobacterium Bovis* infection is likely high, the exact rate remains unknown in Tunisia. Improving molecular diagnostic tools would likely resolve this issue. The control of bovine tuberculosis, which is endemic in Tunisia, is crucial to reducing the incidence of human tuberculous lymphadenitis secondary to *Mycobacterium Bovis* strains. ^11^

### Statistical analysis

In our study, neither age nor gender was predictive of any particular outcome. Similarly, comorbidities did not appear to influence the progression of the disease. Seok et al. reported a significant correlation between younger age and residual lymph node enlargement (defined as lymphadenopathy greater than 10 mm in diameter with enhancement patterns of tuberculosis after six months of treatment). ^19^

Furthermore, no significant association was found between the initial size of the lymph node and disease progression. This contrasts with the literature, which associates a size greater than 3 cm with unfavorable outcomes. ^21– 23^ This discrepancy could be explained by measurement errors or sampling issues. An initial lymph node larger than 3 cm may not be associated with poor progression if it was surgically removed during the initial diagnostic procedure.

Pathological skin changes were associated with an unfavorable progression. In contrast, Soriano et al. found that skin changes did not influence prognosis. ^18^

Regarding radiological data, our study revealed that necrosis on CT scans increased the risk of unfavorable disease outcomes. Zhang et al. also found that a larger area of necrosis was associated with a higher likelihood of poor treatment prognosis. They suggested that necrosis reflects high bacterial virulence, weak immunity, or severe allergic responses, and that its increase is associated with poor response to drug therapy. ^24^

A statistically significant correlation was observed between surgical drainage of a cold abscess and unfavorable disease progression. This could be explained by the fact that cold abscesses may form distally from the original persistent lymphadenopathy. However, initial neck dissection was associated with a favorable outcome.

Liu et al. suggested that surgical removal of cervical lymph node tuberculosis could shorten the time for anti-tuberculosis drug treatment. ^25^ Tahiri et al. found that biopsy with subtotal excision was associated with an unfavorable outcome. ^26^

Surgical treatment remains debated in tuberculous lymphadenopathy. Although often difficult and risky, many studies support the theory that surgical treatment should be combined with medical treatment to manage lymph node tuberculosis. ^22, 25, 26^ Lekhbal et al. reported an association between lymphadenopathy greater than or equal to 3 cm and the need for surgery in the treatment of cervical lymph node tuberculosis. ^21^

Further studies are needed to evaluate the effectiveness of surgical treatment and establish standardized surgical protocols for lymph node *tuberculosis.*

Prolonged ethambutol therapy beyond the first two months was statistically significantly associated with a favorable disease outcome. This supports the hypothesis that *Mycobacterium Bovis* strains are frequently involved in tuberculous lymphadenopathy in Tunisia. ^9, 11^

In our study, improvement at the end of the second month of treatment was significantly associated with a favorable progression. The two-month evaluation may predict the overall outcome and help clinicians manage the disease by conducting further investigations or modifying the therapeutic approach.

### Limitations

The main limitation of the present study was its retrospective design. Additionally, the study was conducted during the COVID-19 pandemic, which led to the postponement or omission of many complementary exams.

Furthermore, our study was based on a sample that may not be representative of the entire population affected by cervical lymph node tuberculosis, especially since HIV-positive patients are followed in another department. Both microbiological and molecular diagnoses were insufficient. The surgical procedures performed for initial diagnosis were not uniform across all patients, making it difficult to compare outcomes.

In our study, a 9-month period was used to define the outcome; however, in most published studies focusing on outcomes, this period was set at 6 months.

## Conclusion

In our study, lymph node dissection and prolonged ethambutol therapy were the main factors associated with favorable outcomes. Our findings underscore the crucial role of surgery in managing lymph node tuberculosis and highlight the importance of considering regional epidemiological factors of tuberculosis when bacteriological or molecular diagnosis is unavailable.

Further research is needed to enhance our understanding of lymph node tuberculosis and to better standardize both medical and surgical treatments, particularly in countries where bovine tuberculosis is endemic.

## Ethical approval: Haut du formulaire

This study was approved by the Ethics Committee of Rabta Hospital (Approval number: CERB 04/2025, approved on February 25, 2025). Given the retrospective and descriptive nature of the study, informed consent from participants was not required, as per the ethics committee’s approval. All data were fully anonymized prior to analysis to ensure confidentiality, in compliance with ethical standards, and in adherence to the Declaration of Helsinki.

## Data Availability

Zenodo: *Factors Predicting Outcome in Cervical Lymph Node Tuberculosis: Insights from a Tunisian Case Series,* https://doi.org/10.5281/zenodo.15351592 ^27^ This project contains the following underlying data: Data file 1. Study Database.sav Data file 2. Table -1-.pdf. The data is available under the terms of the Creative Commons Attribution Non-Commercial No Derivatives 4.0 International (CC BY-NC-ND 4.0) license. Table 1: Univariate analysis of factors influencing the outcome. Table 1 is available as extended data on Zenodo at https://zenodo.org/records/15351592. DOI: 10.5281/zenodo.15351592
