# Supplementary material for: Factors predicting outcome in cervical lymph node tuberculosis: insights from a Tunisian case series
Source: F1000Res. 2025 Sep 30;14:511. Originally published 2025 May 21. [Version 2] doi: 10.12688/f1000research.164097.2 (PMC12498516; doi:10.12688/f1000research.164097.2)
Supplement: Peer review history

# Reviewer report: Reviewer response for version 2

**DOI:** 10.5256/f1000research.188524.r419255

**Published:** 2025-10-01

**Authors:** Ma Jalil Chowdhury, M Mainul Hasan Chowdhury Lt. Col. Chowdhury Lt. Col.

**Affiliations:** 1 Combined Military Hospital, Ramu Cantonment, Bangladesh; 2 CMH, Dhaka, Bangladesh, Dhaka, Dhaka, Bangladesh

**Study design:** Diagnosing of tuberculous lymphadenitis based only on histopathological findings may create diagnostic dilemma particularly when the full findings are not visible in the report. The author here in this paper did not mention on what histopathological findings the diagnoses were made.

**Evaluation:** Evaluation depending upon duration of treatment is often misnomer because of the fact that often the concerned physician for lymph node tuberculosis extends the duration of treatment on empirical basis. Evaluation on the size of lymph node is again misnomer. Because non-regression of the size doesn’t always mean non responding. In case of lymph node tuberculosis, the size may not decrease even may increase during or even at the end of treatment because of paradoxical reaction (PR) which is peculiar to anti-TB therapy in lymph node TB. Extending the duration of treatment beyond 6 months solely depending on non-regression or increase in the size of lymph node is not justifiable. Recurrence or resistant TB should not be based on repeat histopathological findings but should be based only on findings of Mycobacterial tuberculosis on culture and even NOT GeneXpert. Because GeneXpert may be positive on dead bacilli. Conventional histopathological findings may persist even after completion of 6- month ant-TB therapy because of delayed hypersensitivity reaction against tuberculo-protein of dead bacilli.

**Results and Discussion: **There should have been scope for diagnosing Bovin TB Lymphadenitis in those who consumed raw milk. Otherwise, the data regarding consumption of raw milk is not useful. It is not understood whether the cutaneous sinus tract was present following doing FNAC which is quit common phenomenon. FNAC was performed in 85 patients. How others were diagnosed? Epithelioid granuloma with necrosis was observed in 33 patients. How others were diagnosed? Microbiological testing was done in 21 patents i.e. 20.6 %; the question is how the percentage was calculated? However, this is meagre for a scientific presentation. Molecular testing was carried out in 21 patients. The question is which 21? Finally, what were the basis of diagnosis of tuberculous lymphadenitis in the case series? If it is solely based on histopathological examination, it will be questionable. Intermediate resistance to rifampicin was found in 7 patients; so, question is how they were treated and what was their outcome? Was microbiological testing done on surgical samples? What was the basis of second-line antituberculosis drugs in 11 patients? Tuberculous recurrence was observed in 12 patients? How the recurrence was diagnosed? Without microbiological testing, it will not be acceptable.

 Prolonged ethambutol therapy hypothetically thinking of bovine tuberculosis without confirmation may give wrong information to the reader. Intact skin cannot be considered as indicator for favourable outcome. Because deep seated lymph node may lead to formation of sterile abscess in future because of inadequate drainage and may give rise to recurrences and may need adequate surgical clearance.

Is the work clearly and accurately presented and does it cite the current literature?

Partly

If applicable, is the statistical analysis and its interpretation appropriate?

I cannot comment. A qualified statistician is required.

Are all the source data underlying the results available to ensure full reproducibility?

Yes

Is the study design appropriate and is the work technically sound?

Partly

Are the conclusions drawn adequately supported by the results?

Partly

Are sufficient details of methods and analysis provided to allow replication by others?

Partly

Reviewer Expertise:

NA

We confirm that we have read this submission and believe that we have an appropriate level of expertise to confirm that it is of an acceptable scientific standard, however we have significant reservations, as outlined above.

# Reviewer report: Reviewer response for version 1

**DOI:** 10.5256/f1000research.180548.r406892

**Published:** 2025-09-17

**Authors:** Sufian Zaheer

**Affiliations:** 1 Vardhman Mahavir Medical College and Safdarjung Hospital, New Delhi, New Delhi, India

Please elaborate cytopathological findings in your results and tabulate it. It will be very helpful.

 You may use the following articles pertinent to your research in discussion regarding cytopathological findings. 

 1. Kumari A, et al 2023 [Ref 1]

 2. Ahuja S, et al., 2024 [Ref 2]

3. Ahuja S, et al., 2024 [Ref 3]

 Introduction

 -- Could include a more detailed review of previous Tunisian data for comparison.

 Mateial and results:

 -- why Microbiological confirmation performed in few cases (20.6%).

 --- Why were HIV-positive cases excluded

 -- Using **9 months instead of 6 months** as cutoff may reduce comparability with other studies.

 --- Small sample size and low bacteriological confirmation limit generalizability.

 Discussion

 Please discuss latest research in the field including as suggested

Is the work clearly and accurately presented and does it cite the current literature?

Partly

If applicable, is the statistical analysis and its interpretation appropriate?

Partly

Are all the source data underlying the results available to ensure full reproducibility?

Partly

Is the study design appropriate and is the work technically sound?

Partly

Are the conclusions drawn adequately supported by the results?

Yes

Are sufficient details of methods and analysis provided to allow replication by others?

No

Reviewer Expertise:

Cytopathological aspect of infectious disease

I confirm that I have read this submission and believe that I have an appropriate level of expertise to confirm that it is of an acceptable scientific standard, however I have significant reservations, as outlined above.

## References

[1] : Cytomorphological findings in drug defaulters of tuberculous lymphadenitis. *Cytojournal* .2023;20: 10.25259/Cytojournal_16_2023 10.25259/Cytojournal_16_2023 37810436 PMC10559488

[2] : Tuberculous parotitis: A case series diagnosed on fine needle aspiration. *IDCases* .2024;38: 10.1016/j.idcr.2024.e02112 10.1016/j.idcr.2024.e02112 PMC1158460039582745

[3] : Cytomorphological findings in confirmed cases of tubercular lymphadenitis. *Cytojournal* .2024;21: 10.25259/Cytojournal_98_2023 10.25259/Cytojournal_98_2023 38841416 PMC11152538

# Response

**Published:** 2025-09-17

**Authors:** Sufian Zaheer

**Affiliations:** Medical College, India

My concerns are not met in the article

# Response

**Published:** 2025-09-19

**Authors:** Maissa Lajhouri

**Affiliations:** Otorhinolaryngology, Rabta Hospital, Tunis, Tunis, Tunisia

**Comments 1 and 6:** Please elaborate cytopathological findings in your results and tabulate it. It will be very helpful. You may use the following articles pertinent to your research in discussion regarding cytopathological findings.

1. Kumari A, et al 2023 [Ref 1]
2. Ahuja S, et al., 2024 [Ref 2]
3. Ahuja S, et al., 2024 [Ref 3]

**Response:**

 We have elaborated on the cytopathological findings in the Results section and provided a table summarizing the different patterns observed. Additionally, the suggested references regarding cytopathology in tuberculosis have been reviewed and incorporated into the Discussion section.

**Comment 2:** Introduction – Could include a more detailed review of previous Tunisian data for comparison.

**Response:**

 Thank you for your valuable comment. We have expanded the Introduction to include a more detailed review of available data on lymph node tuberculosis in Tunisia. Your comment also allowed us to correct an error in a sentence reporting tuberculosis incidence (in the Discussion section) : Tunisia is a country with intermediate tuberculosis incidence, ranging from 50 to 99 cases per 100,000 population per year, according to the 2023 WHO Report.

**Comment 3:** Material and results – Why was microbiological confirmation performed in only a few cases (20.6%)?

**Response:**

 Microbiological confirmation was performed in only 20.6% of cases, as these were the only results available in the patients’ records. Moreover, in most cases, only cytological examination was performed after fine-needle aspiration, and the aspirated material, including rinse fluid, was not routinely sent for bacteriological testing. Molecular testing was also rarely performed, likely due to the inconsistent availability of PCR kits during the study period.

**Comment 4:** Why were HIV-positive cases excluded?

**Response:**

 HIV-positive patients were not intentionally excluded; they are routinely followed up in a separate department (Department of Infectious Diseases) at our institution. Tunisia has a low HIV incidence according to the WHO, although it has increased in recent years. To address this point, we added a statement in the Discussion section recommending HIV testing in cases of tuberculous lymphadenitis, particularly in patients with unfavorable outcomes.

**Comment 5:** Using 9 months instead of 6 months as cutoff may reduce comparability with other studies.

**Response:**

 Although WHO and other expert society guidelines recommend a 6-month regimen, a 9-month treatment course is still commonly adopted by some practitioners for tuberculous lymphadenitis. This approach is justified by the persistence of large lymph nodes after 6 months, likely due to limited drug penetration. Because of the retrospective nature of our study, we considered both 6- and 9-month treatment durations when defining favorable outcomes.

**Comment 7:** Small sample size and low bacteriological confirmation limit generalizability.

**Response:**

 We have added a statement in the Conclusion section acknowledging that the small sample size and low rate of bacteriological confirmation limit the generalizability of our findings.

# Reviewer report: Reviewer response for version 1

**DOI:** 10.5256/f1000research.180548.r399070

**Published:** 2025-09-12

**Authors:** Vatsal Bhushan Gupta

**Affiliations:** 1 Pulmonary Medicine, City Superspeciality hospital, Gorakhpur, Uttar Pradesh, India

Overall well written. The topic has been described in detail as there are lot of grey areas in management of cervical lymph node TB.

 Certain queries which need to be resolved.

 1. Labelling of figure 1

 2. Current treatment of DSTB is 2 months of HRZE and 4 months of HRE. Why was ethambutol given only for 2 months in most cases

 3. Do you conclude that surgical treatment should be carried out in all cases of lymph node TB as some cases may be managed by medical therapy once the diagnosis is confirmed

Is the work clearly and accurately presented and does it cite the current literature?

Yes

If applicable, is the statistical analysis and its interpretation appropriate?

Yes

Are all the source data underlying the results available to ensure full reproducibility?

Yes

Is the study design appropriate and is the work technically sound?

Yes

Are the conclusions drawn adequately supported by the results?

Partly

Are sufficient details of methods and analysis provided to allow replication by others?

Yes

Reviewer Expertise:

COPD, ILD, sleep disorder

I confirm that I have read this submission and believe that I have an appropriate level of expertise to confirm that it is of an acceptable scientific standard.

# Response

**Published:** 2025-09-19

**Authors:** Maissa Lajhouri

**Affiliations:** Otorhinolaryngology, Rabta Hospital, Tunis, Tunis, Tunisia

**Comment 1:** Labelling of figure 1

**Response:**

 We have completed and clarified the labelling of Figure 1.

**Comment 2:** Current treatment of DSTB is 2 months of HRZE and 4 months of HR. Why was ethambutol given only for 2 months in most cases?

**Response:**

 According to WHO guidelines and official Tunisian recommendations, the standard regimen for drug-susceptible tuberculosis consists of 2 months of HRZE followed by 4 months of HR. A Maghrebian recommendation published in 2015, taking into account the high prevalence of *M. bovis*, suggests 2 months of HRZE followed by 4 months of HR, with ethambutol optionally extended for 1 to 4 additional months beyond the initial 2 months if clinically indicated. This approach was applied in our series based on local practice patterns and patient-specific considerations.

**Comment 3:** Do you conclude that surgical treatment should be carried out in all cases of lymph node TB, as some cases may be managed by medical therapy once the diagnosis is confirmed?

**Response:**

 We do not recommend systematic surgical treatment for lymph node tuberculosis. The decision for surgery should be made on a case-by-case basis. When lymph nodes are small, multiple, or bilateral, surgery should generally not be part of first-line management. A 2-month treatment evaluation is particularly helpful to assess response and guide the need for surgery. Surgery may be proposed in cases showing no improvement or enlargement of lymphadenopathies despite adequate medical therapy. When surgery is performed, it is preferable to remove the maximum number of affected lymph nodes safely, while carefully considering the risk of adhesions.
